# Prevalence and correlates of job loss among schizophrenia outpatients at St. AmanuelMental Specialized Hospital, Addis Ababa, Ethiopia; cross sectional study

**DOI:** 10.1371/journal.pone.0242352

**Published:** 2020-12-28

**Authors:** Yohannes Kifle Chafi, Tadele Amare, Kelemua Haile, Woynabeba Damene, Getaneh Tesfaye, Woredaw Minichil

**Affiliations:** 1 Department of Psychiatry, College of Health Science, Aksum University, Axum, Ethiopia; 2 Department of Psychiatry, College of Medicine and Health Science, University of Gondar, Gondar, Ethiopia; 3 Research and Training Department, St. Amanuel Mental Specialized Hospital, Addis Ababa, Ethiopia; University of Toronto, CANADA

## Abstract

**Background:**

Job loss in patient with chronic illness like schizophrenia is the most serious public concern in the clinical and socio-economic terms worldwide. Patients with schizophrenia usually have unsatisfactory job termination like quitting or getting fired which results well-established negative outcomes. The complex interplay between one another has made job and mental illness the focus areas. In the developing nations, there is limited study on these areas in spite of higher rates of job loss.

**Objectives:**

This study aimed to assess the prevalence and correlates of job loss among schizophrenia outpatient units in Addis Ababa, Ethiopia.

**Methods:**

Institutional based cross-sectional study was conducted at Saint Amanuel Mental Specialized Hospital among schizophrenia outpatient units in Addis Ababa from May to June 2018. A total of 421 study subjects were interviewed using Positive and Negative Syndrome Scale and Perceived devaluation and discrimination scale. Out of the total study participants, female populations were slightly higher (50.4%) and the majorities (38.6%) were orthodox religion followers. Study characteristics was summarized using descriptive statistics and bi-variable and multivariable analysis was performed using Statistical Package for Social Science version 24. Furthermore, those factors at p value ≤ 0.05 were considered as statistically significant.

**Results:**

The prevalence of job loss among patients with schizophrenia was 37.3%. Factors including unmarried [AOR = 2.42:95% CI (1.28, 4.54)], divorced [AOR = 2.34: 95% CI (1.16, 4.71)], severe positive symptoms [AOR = 2.03: 95% CI (1.15, 3.60)], severe general psychopathology [AOR = 1.76: 95% CI (1.01, 3.08)], and poor level of social and occupational functioning [AOR = 5.05: 95% CI (2.81, 9.09)] were significantly associated with job loss among schizophrenia people.

**Conclusion:**

This study suggested that job loss among schizophrenia outpatients was high. There was significant association among people with unmarried, divorced, severe positive symptoms, poor functionality and higher general psychopathology. Therefore, clinical and psychosocial factors were responsible for job loss which warrant further attention and investigation.

## Introduction

Schizophrenia is severe mental illness characterized by two or more abnormalities of delusion, hallucination, disorganized thinking or speech, grossly disorganized or abnormal motor behavior and negative symptom [1]. It is one of the most debilitating disease displaying a wide range of disability in both cognitive domains and everyday functioning [2]. Job is any work that an individual get paid for [3]. Job loss is defined as voluntary or involuntary termination or layoff from one’s occupation because of ill health whereas involuntary job loss is an early retirement before the scheduled or regular retirement age [4].

Schizophrenia is one of the most common of the serious mental disorders and sometimes referred to as a syndrome, the group of schizophrenias having lifetime prevalence of 1%. It is ranked by WHO as the ninth most disabling condition [1, 5]. Career disruption is extensive among people with schizophrenia, even for those who attain job; the length of employment tends to be relatively short, less than 1 year [6]. Generally health related job loss in United Kingdom accounts about 13% through marked increment among mental disorders and musculoskeletal problems [7]. National survey of people with psychotic disorders found that only 13.3% of people with schizophrenia maintained employment for 6 months or more [8]. The negative effect job loss is well established on patients as evidenced by the first author on employment status. Patients who are job losers lack the opportunity that job maintainers possess like an integrated social life, improved symptom level, good quality of life, and better self-esteem [9, 10]. Job loss is a consequence of long-term unemployment which is associated with greater incidence of suicide [11]. The overall outcomes of job insecurity end up with intra-personal and inter-personal disruption, loss of function and poor quality of life [7, 12].

It is well known that schizophrenia plays a role to exit from paid employment, due to disability and early retirement and the main pathway of leaving the labor force differs, particularly between the involuntary and more voluntary routes such as early retirement [13, 14]. Despite these huge impact, patients with schizophrenia are facing not only lack of choice in employment and work opportunity, but also they have financial penalty of working, stigma and discrimination and loss of health benefits [15]. The overall employment gap for severe mental illness is estimated to be around 40 percent [16]. Up to 70 per cent of people with severe mental illness express a desire to work [17]. The complex interplay between one another has made mental health and work the major areas for policy makers and consequently various models of supported job in most cases described as gradualistic approaches to rehabilitation is practiced in some nations [4, 18, 19].

Job loss in schizophrenia outpatient units is highly increasing because of the standard approach of employment system taken today is to discharge from membership. A cohort study in London reports a high initial employment rate fall from 65% to 49% at 2 years [20]. It was 27% in the longitudinal study of West Birmingham UK [21], 44.2% in the cross sectional study of Poland [22] and 40.7% in china [23]. Some studies in employment status and schizophrenia identified the possible associated factors which were; co-morbidity comprising mental and physical illness [24], Occupational type [21], age of onset of illness [22], being male [25], being unmarried [16, 26], low level of education [27], and older age [22, 23, 28], poor social skills [25, 29, 30], severity of symptoms and perceived stigma [31]. Job status comprises multidimensional role in almost all aspects of life among each individual patient. Therefore, this study aimed to assess the prevalence and correlates of job loss in people with schizophrenia which thereafter tackled the proposed objectives.

## Methods and materials

### Sample

An institution based cross-sectional study was conducted among patients diagnosed with schizophrenia at St. Amanuel Mental Specialized Hospital (SAMSH) in Addis Ababa, the capital city of Ethiopia, from May to June 2018. Patients with schizophrenia in St. Amanuel Mental Specialized Hospital have been served for longer period from months to decades. Those patients who used to do their job before the onset of the illness fulfilled the inclusion criteria and those who had never held job and unable to communicate were excluded. Systematic random sampling technique was employed to recruit a total 421 study participants. The sampling fraction (k) was determined by dividing the average number of patients with schizophrenia who had monthly follow-up service to the calculated sample size, which was 7. The first participant was chosen randomly by lottery method from numbers 1 to 7. Thus, those follow up patients who held job before the development of schizophrenia was invited in every seventh to detect weather job loss has occurred or not due to the onset of illness and the associated factors as well.

#### Ethics statement

The study got an ethical approval from the institutional review board (IRB) of University of Gondar and St. Amanuel Mental Specialized Hospital. Full written informed consent was ensured from all participants of people with schizophrenia. The ethics committee specifically approved the questionnaires and the methods of gaining consent. This was considered ethically appropriate as the study was cross sectional one with minimal risk.

### Sample size determination

The sample size was calculated based on single population proportion formula as follow.

$$n={Z_{\frac{\infty }{2}}}^{2}*\frac{P(1-P)}{d^{2}}=384$$

Where,

p (population proportion) = 50%

d (margin of error) = 5%,

${Z_{\frac{\infty }{2}}}^{2}$ (the standard normal distribution and is based on a 95% confidence limit) = 1.96

Assuming 10% non-respondent rate, the total sample size was **423.**

### Variables

Dependent variable was job loss (yes/ no)

Independent variables include socio-demographic variables like age, gender, marital status, ethnicity, work history, educational status, and living condition; clinical variables like symptom severity, age of onset, duration of illness, number of hospitalizations, number of episode/relapses, and vocational training, co morbidity and psychosocial variables including social support and stigma; level of social and occupational functioning.

### Measurement

Job loss was confirmed by yes/ no questions of whether previously working schizophrenia patients maintain or lose their job due to the onset of the illness either voluntarily or involuntarily.

Positive and Negative Syndrome Scale (PANSS) was used for symptom severity assessment which was first devised and published in 1987 by Stanley Kay, Lewis Opler, and Abraham Fiszbein [32]. The scale is well known and widely used instrument that all assessments of psychotic behavioral disorders should follow. It is 30 item scale that has three subscales; positive symptom scale, negative symptom scale and general psychopathology scale [33, 34]. Social and occupational functioning assessment scale (SOFAS) was used to assess patient’s level of social and occupational function which was measured as “poor” and “good” from a cut point of 60. It measures the level of functional impairment due to both physical and mental illness independent of symptoms [35, 36]. Oslo Social Support Scale defines Poor social support (”3–8” score), Moderate social support (9–11 score), Strong social support (12–14) [37]. Perceived devaluation and discrimination scale (PDDS) was used to evaluate the level of perceived stigma which was scored as low and high with the cut point of mean score [38].

### Data collection procedure

Semi-structured questionnaires were used to collect the relevant data including yes/no questions, socio-demographic, illness related, and psychosocial characteristics questions. Four trained psychiatric nurses participated in the data collection and data collectors were given timely supervision. The questionnaire was designed in English and translated from English to Amharic, local and official language of Ethiopia, and back to English to keep its consistency. Those patients who had held job during first diagnosis of schizophrenia was invited as study subjects.

### Data quality assurance

Emphasis was given for the quality of the data by using different strategies. Training was given for the data collectors and supervisors on the techniques of data collection, ethical principles of research and interviewing techniques. The questionnaire was pretested among 21 samples obtained from Saint Paul Millennium Hospital in Addis Ababa before the actual data collection period. The questionnaire was well checked for clarity, simplicity, and understandability. During the data collection period, each items of the questionnaire were checked for completeness on daily basis by the data collectors and supervisors.

### Data processing and analysis

The coded variables were entered to Epi-data version 3.1 and then exported to SPSS (Statistical Package for Social science) version 24 for analysis. Descriptive statistics was used to summarize the distribution of the variables. Binary logistic regression was carried out to see the associations between job loss and each independent variable. Variables with p-value < 0.2 in the bivariate logistic regression were entered into multivariable logistic regression model for further analysis. Those factors at P-value ≤ 0.05 using adjusted odds ratio with 95% confidence level were considered as statistically significant. Model fitness, using Hosmer and Lemshow test, was checked.

## Ethical consideration

Ethical approval for the study was obtained from the joint Ethical Review Board (ERB) of University of Gondar College of Medicine and Health Science and St. Amanuel Mental Specialized Hospital. After explaining the purpose of the study, written informed consent was obtained from each participants of people with schizophrenia. Confidentiality was maintained by omitting their personal identification.

## Results

Total of 421 participants with 99.5% response rate were included in this study. Before the onset of the illness job status of most of people with schizophrenia was trade persons 42.5% (179) followed by government employees 24.2% (102), farmers 14.7% (62) and other jobs like maid, labor force and brokers about 18% (78).

### Socio-demographic characteristics of the study participants

Out of the total participants nearly half 50.4% (212) were females. The mean age of the respondents was 35.8% ± SD 9.08 years. Regarding ethnicity 30.6% (129) were Oromo and 36.8% (155) were Orthodox followers in religion. In terms of marriage 39.2% (165) of the participants had never married and towards the educational level 31.6% (133) completed primary school (Table 1).

**Table 1:** Socio-demographic characteristics of patients with schizophrenia at St. Amanuel Mental Specialized Hospital, Addis Ababa, Ethiopia, 2018 (n = 421).

Variable	Category	Frequency (N)	Percent (%)
**Sex**	Male	209	49.6
	Female	212	50.4
**Age**	18–24	20	4.8
	25–35	192	45.6
	≥36	209	49.6
**Marital status**	Married	104	24.7
	Unmarried	165	39.2
	Separated	70	16.6
	Divorced	82	19.5
**Religion**	Orthodox	155	36.8
	Muslim	130	30.9
	Protestant	112	26.6
	Catholic	24	5.7
**Ethnicity**	Amara	79	18.8
	Oromo	129	30.6
	Tigrai	92	21.9
	Guragie	96	22.8
	Siltie	25	5.9
**Living arrangement**	Alone	46	10.9
	Family	323	76.8
	Friend	37	8.8
	Relatives	15	3.6
**Level of education**	Illiterate	59	14
	Primary school	133	31.6
	Secondary school	129	30.6
	Above diploma	100	23.8
Job status	Gov’t employ	53	12.6
	Trade person	117	27.8
	Farmer	46	10.9
	Daily labor	48	11.4
	Job loss	157	37.3

### Clinical characteristics of the study participants

Regarding the clinical characteristics of participants 48.2% (203) had age of onset in the late twenties and early thirties (26–35) years and majorities of the respondents (80.5%) had recurrent episode of illness. More than half of the participants 51.8% (218) had up to three (1–3) times admission history, concerning the total duration of illness 45.6% (192) respondents had up to 10 years’ of length of illness, and nearly half 50.4% (212) of the total study subjects developed severe general psychopathology (Table 2).

**Table 2:** Clinical characteristics of patients with Schizophrenia at St. Amanuel Mental Specialized Hospital, Addis Ababa, Ethiopia, 2018 (n = 421).

Variable	Category	Frequency(N)	Percent (%)
**Onset of illness**	<15	7	1.7
	15–25	178	42.3
	26–35	203	48.2
	≥ 36	33	7.8
**Number of episodes**	Single	82	19.5
	Recurrent	339	80.5
**Number of admissions**	No	167	39.7
	1–3	218	51.8
	>3	36	8.6
**Length of illness**	<5	106	25.2
	5–10	192	45.6
	>10	123	29.2
**Rehabilitation service**	Yes	71	16.9
	No	350	83.1
**Positive symptom**	Mild	255	60.7
	Severe	166	39.3
**Negative symptom**	Mild	217	51.5
	Severe	204	48.5
**General psychopathology**	Mild	209	49.6
	Severe	212	50.4

### Psychosocial characteristics of the study participants

Schizophrenia disorder causes the patient for social and occupational exclusion. In this study, most of the participants 66.7% (278) had poor social and occupational functioning and 43.5% (183) got poor social support (Table 3).

**Table 3:** Social and functional characteristics of patients with schizophrenia at St. Amanuel Mental Specialized Hospital, Addis Ababa, Ethiopia, 2018 (n = 421).

Variable	Category	Frequency (N)	Percent (%)
Social and occupational function	Poor	281	66.7
(SOFAS)	Good	140	33.3
social support level	Poor	183	43.5
	Moderate	187	44.4
	Strong	51	12.1

NB: SOFAS = Social and Occupational Functioning Assessment Scale.

### Prevalence of job loss among schizophrenia outpatients

The study showed that the prevalence of job loss among schizophrenia outpatients at St. Amanuel Mental Specialized Hospital who had had job during first diagnosis was 37.3% (157), (95% CI: 32.8, 42.0). Of these, the majorities (84) were male participants and 27.4% (43) were working in trading and private institution (S1 Fig).

### Logistic regression of factors associated with job loss

In the bivariate analysis of job loss in relation to each explanatory variables; marital status, level of education, type of work in the past, other mental illness, length of illness, number of episode, physical illness, positive and negative symptoms and social and occupational functioning were variables that fulfilled p-value less than 0.2 and then exported to multivariable logistic regression for further analysis.

In the multivariable logistic regression analysis being unmarried and divorced, severity of positive symptoms, severity of general psychopathology, and poor outcome in social and occupational functioning were statistically significant at p value ≤ 0.05.

The odds of job loss among schizophrenia patients who had poor social and occupational functioning was 5 times higher [AOR = 5.05: 95% CI (2.81, 9.09)].

Regarding marital status, the odds of job loss in unmarried schizophrenia patients was 2 times higher than married patients [AOR = 2.42:95% CI (1.28, 4.54)]. Similarly, the odds of job loss in divorced schizophrenia patients was about 2 times higher than married patients [AOR = 2.34:95% CI (1.16, 4.71)].

Furthermore, the odds of job loss among participants who had severe positive symptoms was 2 times higher [AOR = 2.03: 95% CI (1.15, 3.60)]. Additionally, the odds of job loss in patients with severe general psychopathology was 2 times higher than patients with mild general psychopathology [AOR = 1.76:95% CI (1.01, 3.08)] (Table 4).

**Table 4:** Bivariate and multivariable binary logistic regression of factors associated with job loss among patients with schizophrenia at St. Amanuel Mental Specialized Hospital, Addis Ababa, Ethiopia, 2018 (n = 421).

Variable	Category	Job loss		COR (95% CI)	AOR (95% CI)	p-value
		Yes	No			
**Marital status**	Married	26	78	1.00	1.00	
	Unmarried	65	100	1.95(1.13, 3.36)	2.42(1.28, 4.54)	0.006*
	Separated	27	43	1.88(0.98, 3.63)	1.41(0.66, 2.98)	0.376
	Divorced	39	43	2.72(1.46, 5.06)	2.34(1.16, 4.71)	0.018*
**Length of illness**	<5 years	32	74	1.00	1.00	
	5–10 years	69	123	1.30(0.78, 2.16)	0.83(0.45, 1.53)	0.543
	>10 years	56	67	1.93(1.12, 3.34)	1.34(0.69, 2.61)	0.392
**Illness episode**	Single	22	60	1.00	1.00	
	Recurrent	135	204	1.80(1.06, 3.08)	1.27 (0.65, 2.48)	0.489
**Other mental illness**	Yes	26	31	1.49(0.85, 2.62)	0.65(0.33, 1.28)	0.213
	No	131	233	1	1	
**physical illness**	Yes	21	22	1.02(0.90, 3.20)	1.46(0.69, 3.08)	0.327
	No	136	242	1	1	
**Positive symptom**	severe	87	79	2.96(1.99, 4.54)	2.03 (1.15, 3.60)	0.015*
	mild	70	185	1	1	
**Negative symptom**	Severe	89	115	1.70 (1.14, 2.53)	0.77(0.41, 1.42)	0.400
	Mild	68	149	1	1	
**General psychopathology**	Severe	100	112	2.38(1.59, 3.58)	1.76(1.01, 3.08)	0.046*
	Mild	57	152	1	1	
**Social and occupational function**	Poor function	137	144	5.71(3.60, 10.58)	5.05 (2.81, 9.09)	0.001*
	Good function	20	120	1	1	
**Social support**	Poor	73	110	1.59(0.81, 3.12)	0.95(0.58, 1.54)	0.830
	Moderate	69	118	1.40(0.72, 2.75)	0.61(0.28, 1.36)	0.230
	Strong	15	36	1	1	

NB: Model fitness, Hosmer and Lemshow test = 0.89

* = statistically significant at P value ≤ 0.05.

## Discussion

Schizophrenia is one of the chronic mental illness affecting social, occupational and personal areas of functioning. Job loss, job insecurity and lack of work opportunity have characterized the affected populations. Job loss is correlated with poorer physical and psychosocial health in the general population, and it usually worsens in the mentally ill patients.

The prevalence of job loss in this study was 37. 3% (95% CI: 32.8, 42.0). This finding was in line with a study done in china which was 40.7% [23]. The possible reason might be the similarity of study setting, study design, and tools.

The current study was higher than a cohort study done in London which was 16% [20] and in West Birmingham UK which was 27% [21]. The difference in the study design and the total duration of studies might be the cause of the variation.

This study was also higher than an European Schizophrenia Cohort Study in the research centers of German, France and London in which the prevalence of cross national job loss was 8.5% [39]. These might be due to the difference in the study design each used, the total duration of the study and previous job status of the participants in which ours were only those who had job during first diagnosis.

This study was slightly lower than the study done in Poland which was 43.9% [22]. The possible reason might be due to accesses of higher training and job position in developed nations require increased mental functioning that is difficult for schizophrenia patients to maintain their job.

Regarding the associated factors, being unmarried and divorced patients were significantly associated with job loss among people with schizophrenia. Loss of psychosocial support might be the possible reason which was consistent with another cohort study conducted in London [30].

Job loss was also significantly associated with Positive symptom severity and severity of general psychopathology. This might be due to in the severe stage of the illness behavioral and thought disorganization are prominent which affects functional involvement. This was consistent with studies conducted in Poland [22] and the European schizophrenia cohort study (EURO SC) [28].

There was also strong association between occupational and social dysfunction with job loss. This might be due to being alone is the symptom of the illness and failure to involve in the social and occupational areas will speed up the likelihood of job loss. This finding was consistent with another studies done in China [23] and another study in Chicago [40].

## Conclusion

In this study job loss was high among people with schizophrenia. Being unmarried, divorced, positive symptom severity, poor social and occupational functioning and severe general psychopathology were factors significantly associated with job loss. Therefore, clinical and psychosocial factors are mainly involved in causing job loss. These and related factors are important areas for advancing job rehabilitation and maintenance. Job rehabilitation offers the basis for overall quality of life which requires the stakeholders to address these factors and related areas. Effective symptomatic management, occupational and psychosocial support minimize the likelihood of job loss. Further research on these and related factors are valuable.

## Supporting information

S1 Fig Job status of study participants. (TIF)

S2 Fig Conceptual framework that shows the possible factors associated with job loss derived from different literature and books among schizophrenia patients at Saint Amanuel Mental Specialized Hospital, Addis Ababa, Ethiopia, 2018 (n = 421). (PDF)
