# Supplementary figures and images for: Prevalence and correlates of job loss among schizophrenia outpatients at St. AmanuelMental Specialized Hospital, Addis Ababa, Ethiopia; cross sectional study
Source: PLoS One. 2020 Dec 28;15(12):e0242352. doi: 10.1371/journal.pone.0242352 (PMC7769443; doi:10.1371/journal.pone.0242352)

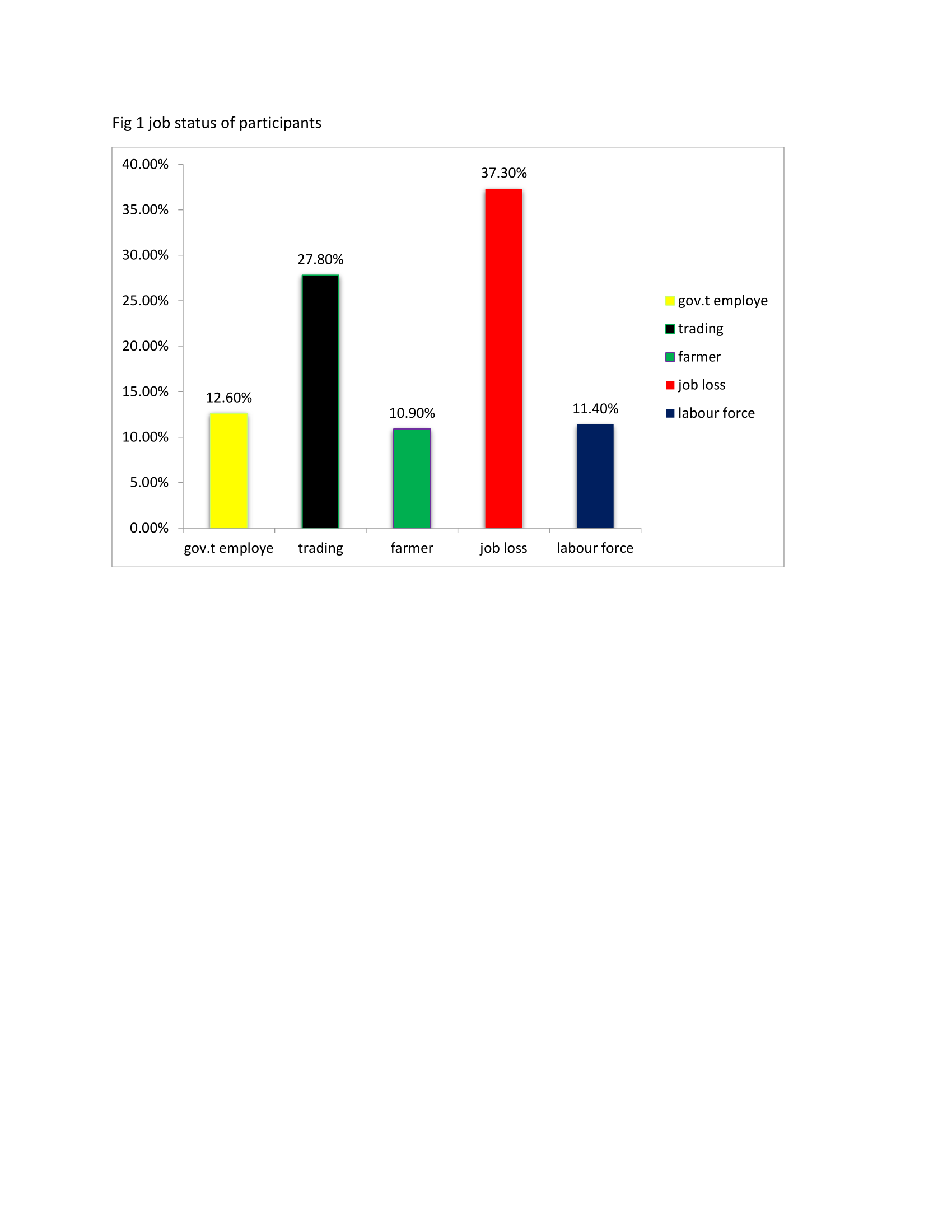

Supplement: S1 Fig — (TIF) [file pone.0242352.s001.tif]
