# Supplementary material for: Prevalence and correlates of job loss among schizophrenia outpatients at St. AmanuelMental Specialized Hospital, Addis Ababa, Ethiopia; cross sectional study
Source: PLoS One. 2020 Dec 28;15(12):e0242352. doi: 10.1371/journal.pone.0242352 (PMC7769443; doi:10.1371/journal.pone.0242352)
Supplement: S2 Fig — (PDF) [file pone.0242352.s002.pdf]

Fig 2 Conceptual frame work

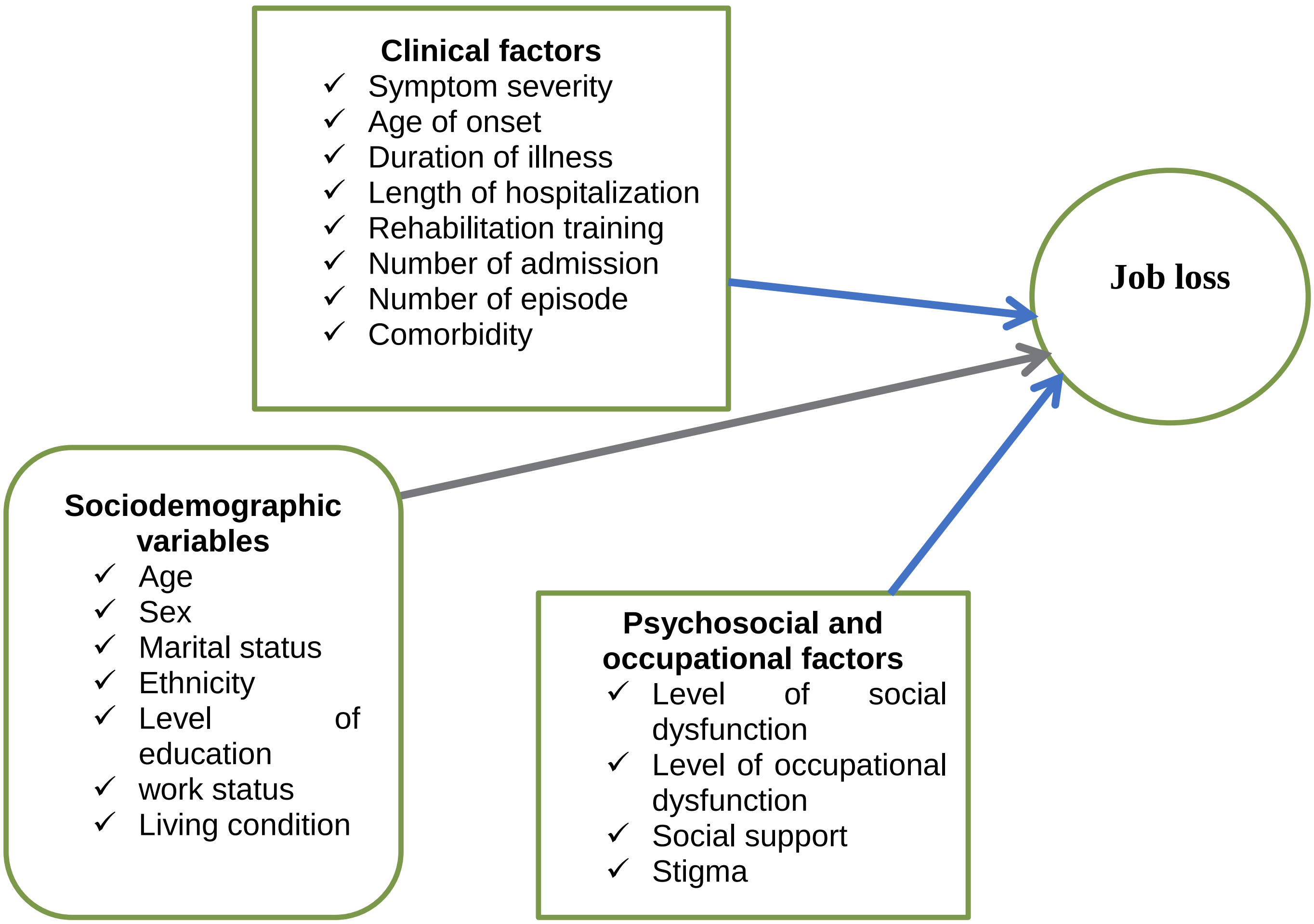

Figure 1: Conceptual framework that shows the possible factors associated with job loss derived from different literature and books among schizophrenia patients at Saint Amanuel Mental Specialized Hospital, Addis Ababa, Ethiopia, 2018 (n=421)
